# Eukaryotic genomes may exhibit up to 10 generic classes of gene promoters

**DOI:** 10.1186/1471-2164-13-512

**Published:** 2012-09-28

**Authors:** Paul Gagniuc, Constantin Ionescu-Tirgoviste

**Affiliations:** 1 Institute of Genetics, University of Bucharest, Bucharest 060101, Romania; 2 National Institute of Diabetes, Nutrition and Metabolic Diseases “N.C. Paulescu”, Bucharest, Romania

**Keywords:** Gene promoters, Promoter classes, Eukaryotic genomes, Promoter patterns, Kappa index of coincidence, Promoter network

## Abstract

**Background:**

The main function of gene promoters appears to be the integration of different gene products in their biological pathways in order to maintain homeostasis. Generally, promoters have been classified in two major classes, namely TATA and CpG. Nevertheless, many genes using the same combinatorial formation of transcription factors have different gene expression patterns. Accordingly, we tried to ask ourselves some fundamental questions: Why certain genes have an overall predisposition for higher gene expression levels than others? What causes such a predisposition? Is there a structural relationship of these sequences in different tissues? Is there a strong phylogenetic relationship between promoters of closely related species?

**Results:**

In order to gain valuable insights into different promoter regions, we obtained a series of image-based patterns which allowed us to identify 10 generic classes of promoters. A comprehensive analysis was undertaken for promoter sequences from *Arabidopsis thaliana*, *Drosophila melanogaster*, *Homo sapiens* and *Oryza sativa*, and a more extensive analysis of tissue-specific promoters in humans. We observed a clear preference for these species to use certain classes of promoters for specific biological processes. Moreover, in humans, we found that different tissues use distinct classes of promoters, reflecting an emerging promoter network. Depending on the tissue type, comparisons made between these classes of promoters reveal a complementarity between their patterns whereas some other classes of promoters have been observed to occur in competition. Furthermore, we also noticed the existence of some transitional states between these classes of promoters that may explain certain evolutionary mechanisms, which suggest a possible predisposition for specific levels of gene expression and perhaps for a different number of factors responsible for triggering gene expression. Our conclusions are based on comprehensive data from three different databases and a new computer model whose core is using Kappa index of coincidence.

**Conclusions:**

To fully understand the connections between gene promoters and gene expression, we analyzed thousands of promoter sequences using our Kappa Index of Coincidence method and a specialized Optical Character Recognition (OCR) neural network. Under our criteria, 10 classes of promoters were detected. In addition, the existence of “transitional” promoters suggests that there is an evolutionary weighted continuum between classes, depending perhaps upon changes in their gene products.

## Background

Promoters have guided evolution for millions of years. It appears that they were the main engine responsible for the integration of different mutations favorable for the environmental conditions [1]. Promoters are critical regions for gene regulation in complex genomes and are located upstream of TSS (Transcription Start Site). A typical promoter region is composed of a core promoter and regulatory domains [2,3]. The structure of a promoter is recognized by the presence of known promoter elements, such as TATA box, GC-box, CCAAT-box, BRE and INR box [4-12]. Therefore, accurate recognition of a promoter structure relies on a comprehensive list of promoter elements. Nevertheless, using these promoter elements for classification has proven to be difficult and perhaps even disadvantageous for different functional correlations between promoter sequences. From an evolutionary standpoint, within non-coding regulatory regions, nucleotides can change their order more frequently and these binding sites often become very small and instable [13]. Previously, approaches towards promoter classification include motif sequences and other structural parameters, such as DNA curvature, bendability, stability, nucleosome positioning or comparison of various DNA sequences [14-19]. Currently, promoters from vertebrates are classified into two major classes, namely TATA and CpG types while in mammals there is a subclassification in TATA box–enriched and CpG-rich promoters [20]. In order to investigate possible interactions between different biological processes, we found that an overall correlation between DNA sequence features among promoter regions may be an alternative method. In this context, we have chosen a different approach to classify promoter sequences by using two-dimensional patterns obtained through Kappa Index of Coincidence (Kappa IC) and (C + G)% values [21-24]. This classification it is mainly done by considering the shape and density of these promoter patterns. In this study, we explore the structural properties of these patterns and we search for correlations between promoter sequences of several different species. Genome sequencing has led to the development of many bioinformatic methods for accurate recognition and extraction of promoter sequences. A number of experimental approaches to compile TSSs on a genome-wide scale have been developed including the Eukaryotic Promoter Database [25,26] and PlantProm Database [27]. We used these databases and focused our attention on 20,597 promoter sequences from *Arabidopsis thaliana*, *Drosophila melanogaster*, *Homo sapiens* and *Oryza sativa*. In humans we were also interested in promoters of genes that are expressed preferentially in certain tissues. Several studies converged on characterizing patterns of tissue specific gene expression, including TiGER (Tissue-specific Gene Expression and Regulation) database [28-30], which contains comprehensive information about human tissue-specific gene expression profiles. We have used TiGER database list of tissue-specific genes to determine the proportion of each promoter class in 30 tissues. This allowed us to identify certain relations between promoter sequences and different biological processes.

## Results

We first investigated if some promoter patterns occur more often then others. Secondly we determined which of these patterns are more common in certain species and whether their distribution may have some evolutionary implications. In the third analysis we examined the distribution of these promoter classes among human tissues.

### Promoter classification

**Figure 1:**
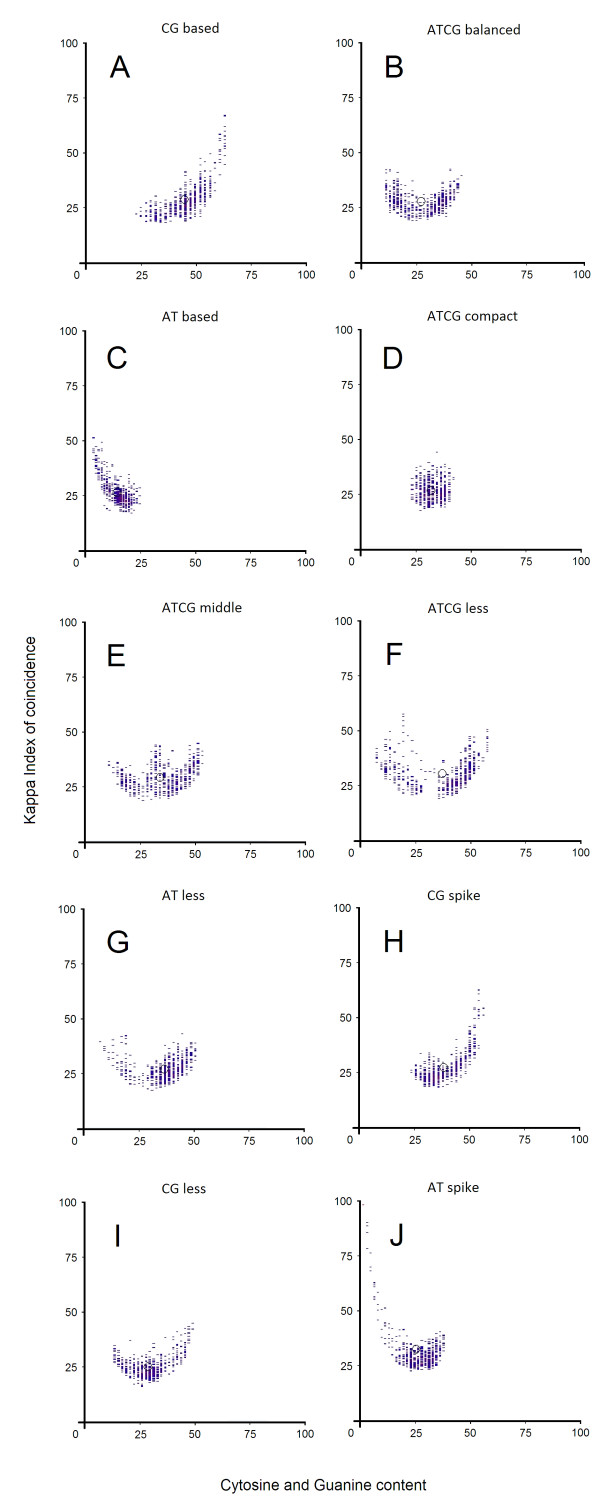
**Ten classes of promoters and their representative patterns.** Each promoter pattern is composed of vertically aligned clusters of Kappa IC (y-axis) and GC% (x-axis) values. The center of weight for each pattern is represented by a black circle. These representative promoter patterns are shown in the following sections as follows: (**A**) AT-based, (**B**) CG-based, (**C**) ATCG-compact, (**D**) ATCG-balanced, (**E**) ATCG-middle, (**F**) ATCG-less, (**G**) AT-less, (**H**) CG-spike, (**I**) CG-less and (**J**) AT-spike.

**Figure 2:**
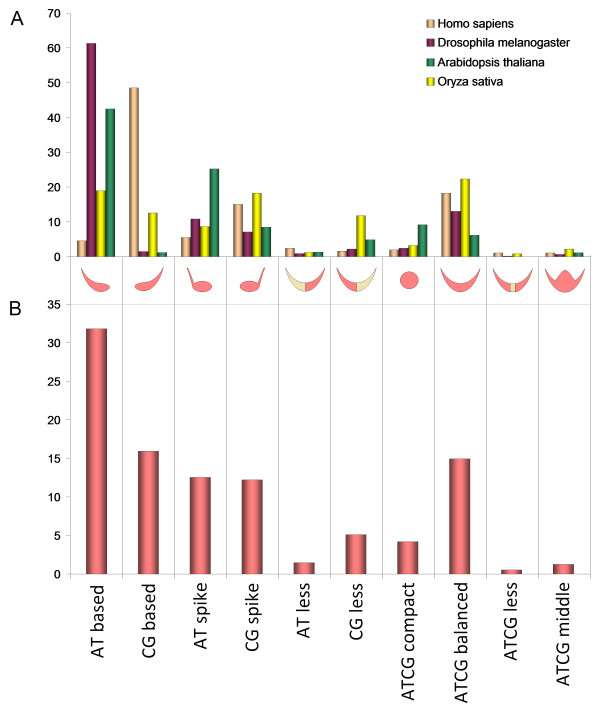
**Organism-specific frequencies of each promoter class.** Each column represents a class of promoters. Starting at the bottom of each column we present the class name, (**B**) the average preference of promoter classes between species, a representative shape of the promoter class (pink areas show denser clusters whereas light grayish gold color shows lower density clusters) and (**A**) the proportion of promoter classes in *Arabidopsis thaliana*, *Drosophila melanogaster*, *Homo sapiens* and *Oryza sativa.*

When promoter patterns are generated, some initial general conclusions can be drawn. Although these promoter sequences are less conserved between species they exhibit similar patterns. Each pattern is composed of vertically aligned clusters of Kappa IC (y-axis) and (G + C)% (x-axis) values. Vertical positions of these clusters form a promoter pattern which has a specific form for each promoter sequence. We have been able to classify promoters according to their patterns and noticed ten general types of promoters (Figure 1A-J). Although the overall shape and density seems to be conserved across different classes of promoters, they do differ in finer details. This may indicate a further possible organization of promoter classes in several subclasses. Their shape is explained by the presence of different structures such as simple sequence repeats (SSRs) or short tandem repeats (STRs). Among these structures we found an interesting distribution of short and long homopolymer tracts or di- and tri-nucleotides formations, many of which are consistent with other studies previously done [31,32]. We have been able to partition these patterns into ten classes on the basis of clear visual distinctions between their shape and their cluster density. The name of each promoter class has been chosen by the average nucleotide content and Kappa IC values, as follows:

1) *AT-based promoters*. AT-based representative patterns are distinguished by high (A + T)% and Kappa IC values. The left side of the pattern is predominant, while the right side is significantly less pronounced. The shape of this pattern exhibits various different lengths of short poly(dA:dT) homopolymer tracts (Figure 1C). AT-based patterns are characteristic for gene promoters from *Drosophila melanogaster* and *Arabidopsis thaliana* and are less common in humans.

2) *CG-based* promoters. These promoters are represented by patterns containing a high percentage of C + G and high Kappa IC values. CG-based promoters show a high CpG content. The right side of the pattern is predominant while the left side is significantly less pronounced (Figure 1A). The shape of this pattern exhibits various different lengths of short poly(dC:dG) homopolymer tracts. In addition, the average frequency of occurrence between AT-based and CG-based promoters appears to differ completely in these species, but curiously, these promoters tend to be in a relative opposition in each species (Figure 2A,B). This observation suggests that these species have different preferences for allocation of certain fundamental functions. Patterns of this class are particulary characteristic for genes from *Homo sapiens*.

3) *ATCG-compact* promoters. ATCG-compact patterns characterize promoters with centrally disposed clusters, leading to the formation of a round shaped pattern (Figure 1D). The middle-lower region of the pattern contains evenly interspersed nucleotides (A,T,C,G ≈ 25%) and the middle-upper area shows different lengths of short homopolymer tracts (poly(dA), poly(dT), poly(dC), poly(dG)) disposed in tandem in any order. ATCG-compact patterns are characteristic for gene promoters from *Arabidopsis thaliana*.

4) *ATCG-balanced* promoters. Promoter sequences belonging to ATCG-balanced class show an almost balanced G + C and A + T content. The right and the left side of the pattern tend to share a relative 2-fold rotational symmetry. These patterns are generally composed of equally distributed short poly(dA:dT) and poly(dC:dG) homopolymer tracts (Figure 1B). ATCG-balanced and CG-spike promoters tend to occur in the same proportion in each species and appear to have almost similar average frequencies between species (Figure 2A,B). This observation indicates that for some specific functions the same classes of promoters are preferred between species. These patterns are characteristic for gene promoters from *Homo sapiens* and *Oryza sativa*.

5) *ATCG-middle* promoters. ATCG-middle patterns are characterized mainly by promoter sequences containing A + T and C + G balanced values and higher than average Kappa IC values. The right side and the left side of the pattern are equally distributed. However, the central part is pronounced. They are similar to ATCG-balanced class in that they also have a relative 2-fold rotational symmetry, but contain additional short homopolymer tracts (poly(dA), poly(dT), poly(dC), poly(dG)) disposed in tandem in any order (Figure 1E). These patterns are rare and are almost equally distributed in all four species.

6) *ATCG-less promoters*. Promoters from this class are represented by an abrupt transition between two C + G threshold levels. Similar to ATCG-balanced promoters, the right side and the left side of the pattern is equally distributed, however, some sequences around the central region are missing or have a lower density. Typically, these central regions lack of tandem short homopolymer tracts and short sequences consisting of equally interspersed nucleotides (A,T,C,G ≈ 25%), or short sequences showing small variations over 50% in favor of A + T or C + G nucleotides (Figure 1F). Based on the promoter sequence features, these promoter patterns seem to be complementary with ATCG-middle promoters. ATCG-less patterns are significantly rare (an overall frequency between species of 0.10% - 0.16%) and are characteristic for promoters from *Homo sapiens* and *Oryza sativa* but are almost absent in *Drosophila melanogaster* and *Arabidopsis thaliana*.

7) *AT-less promoters*. Promoter sequences belonging to AT-less class exhibit a high frequency of short CG-rich sequences. Although both sides of the pattern show a relative 2-fold rotational symmetry, the clusters from the left side of the pattern exhibit a lower density than those on the right. These patterns are characterized by a large number of short poly(dC:dG) tracts and a lower number of short poly(dA:dT) tracts (Figure 1G). Short poly(dA:dT) tracts typically occur as a consequence of an abrupt depletion of C + G nucleotides on short distances (30b–60b) inside the promoter sequence. Such a depletion is accompanied by high Kappa IC values and is typically present near TSS (± 200b), suggesting a regular expression of their genes. AT-less patterns are generally rare and are found equally in all four species, but are slightly more frequent in *Homo sapiens*.

8) *CG-less promoters*. In contrast, CG-less promoters are distinguished by a high frequency of short AT-rich sequences and are more common in *Oryza sativa* and *Arabidopsis thaliana*. The right and left side of the pattern tend to be equally distributed, however, the clusters from the right side of the pattern exhibit a lower density than those on the left. AT-less and CG-less promoters seem to be characterized by an imbalance between the number of short poly(dA:dT) tracts and short poly(dC:dG) tracts. Complementary to AT-less promoter characteristics, these patterns are characterized by a large number of short poly(dA:dT) tracts and a much lower number of short poly(dC:dG) tracts (Figure 1I). Compared with AT-less promoters, the overall preference for CG-less promoters is very high between species. However, in *Homo sapiens* the number of AT-less promoters slightly exceeds the number CG-less promoters (Figure 2A).

9) *AT-spike promoters*. Promoter sequences belonging to AT-spike class are represented by long repetitive sequences with a high content of A or T nucleotides. These patterns exhibit a central part and an elongated left side containing small density clusters. The shape of AT-spike representative patterns is explained by the presence of long poly(dA) or long poly(dT) homopolymer tracts or tandem short poly(dA) or short poly(dT) tracts (Figure 1J). These promoters are prevalent in *Arabidopsis thaliana*.

10) *CG-spike promoters*. In contrast to AT-spike promoter architecture, these promoters are represented by long repetitive sequences with a high content of C or G nucleotides. CG-spike patterns exhibit a central part and an elongated right side containing small density clusters. These patterns contain long poly(dC) or long poly(dG) homopolymer tracts or tandem short poly(dC) or short poly(dG) tracts (Figure 1H). AT-spike and CG-spike promoters seem to be complementary considering the fact that both promoter classes are differentiated by two opposite types of homopolymer tracts. AT-spike and CG-spike classes appear to be equally preferred between species, nevertheless, their promoters tend to be in opposition in each species (Figure 2B). This observation suggests a possible conservation of their antagonist role between these species, yet a different preference for certain functions. These patterns are common in *Oryza sativa* and *Homo sapiens*.

### Promoter distribution

**Figure 3:**
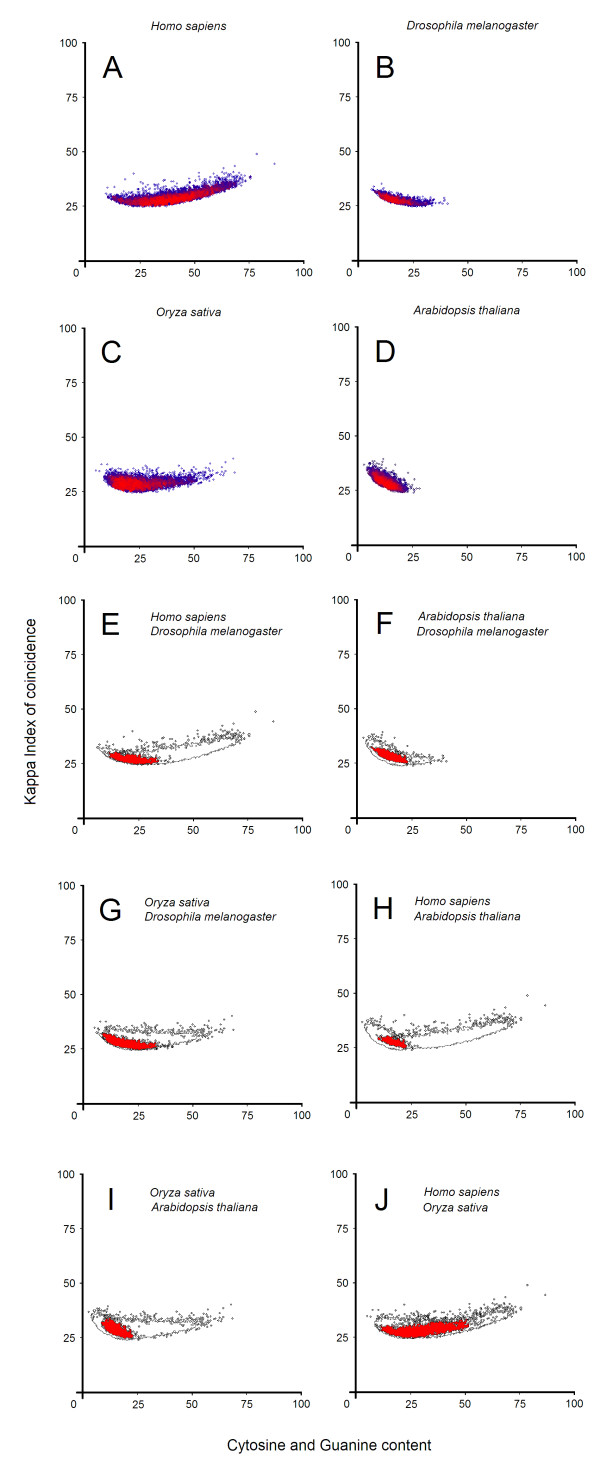
**Promoter distributions for each species.** (**A**) *Homo sapiens*, (**B**) *Drosophila melanogaster*, (**C**) *Oryza sativa* and (**D**) *Arabidopsis thaliana*. Each point represents the center of weight from a promoter pattern. Red color areas represent denser clusters of promoters. (**E-J**) superposition between promoter distributions. Red color areas represent conserved promoter sequences.

**Figure 4:**
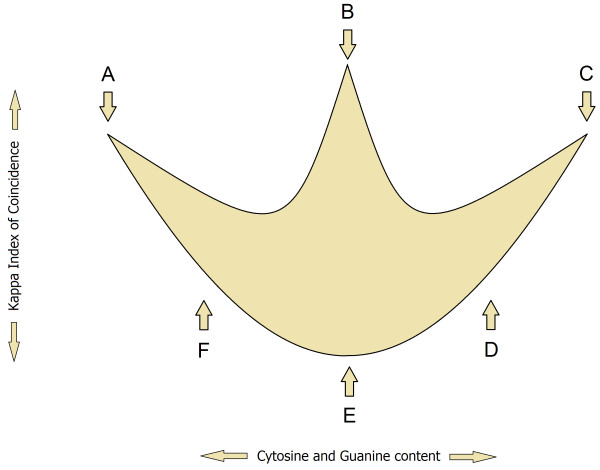
**Location of SSRs and STRs within a promoter pattern.** The light grayish gold shape represents a model of a promoter pattern from ATCG-middle class in which we approximate the location of various structures that compose a promoter sequence. (**A**) long Poly(dA) or Poly(dT) tracts or tandem short Poly(dA) or Poly(dT) tracts, (**B**) non-ordered short Poly(dA) and Poly(dT) and Poly(dC) and Poly(dG) tracts, (**C**) long Poly(dC) or Poly(dG) tracts or tandem short Poly(dC) or Poly(dG) tracts, (**D**) short Poly(dC) and Poly(dG) tracts, (**E**) evenly interspersed nucleotides (A,T,C,G ≈ 25%), (**F**) short Poly(dA) and Poly(dT) tracts.

Our comparative analyses have revealed similarities and differences in the promoter architecture between *Arabidopsis thaliana*, *Drosophila melanogaster*, *Homo sapiens* and *Oryza sativa*. We have plotted the center of weight from 20,586 promoter patterns according with each species in order to highlight the distribution of these regulatory sequences (Figure 3). The center of weight of each promoter pattern indicates an average between all SSR and STR sequences. ATCG-middle patterns contain almost all types of SSR and STR sequences and can reveal some visual insights into different promoter regions (Figure 4A-F). Although the phylogenetic relationships are usualy based on sequence alignment algorithms, Kappa IC approach is based on a frequency/content comparison. A superposition between promoter distributions from each species shows the shared surfaces, representing conserved promoter sequences (Figure 3E-J). Promoter sequences from *Arabidopsis thaliana* and rice were notably differentiated, and only a small part of promoters were shared (Figure 3B,D and Figure 3I). Moreover, *Arabidopsis thaliana* promoters seem to have more structural features in common with those from *Drosophila melanogaster* (Figure 3F). Promoters from *Arabidopsis thaliana* exhibit higher Kappa IC values than promoters from *Drosophila melanogaster*, while variations of C + G content are relatively the same. Curiously, the highest rate of conserved promoters was encountered between *Homo sapiens* and *Oryza sativa* (Figure 3J) and the lowest rate of conservation was observed between *Arabidopsis thaliana* and *Homo sapiens* (Figure 3H). Promoter sequences from *Homo sapiens* show both a wider distribution of C + G content and the highest values of Kappa IC (Figure 3A,E,H,J). The superposition of promoter distributions of the four species shows that promoters do not reflect distant phylogenetic relationships (Figure 3E-J). We have also noticed the directions and the angles of these promoter distributions which may suggest an evolutionary tendency for each species.

### TATA-less and TATA-containing correlations

**Figure 5:**
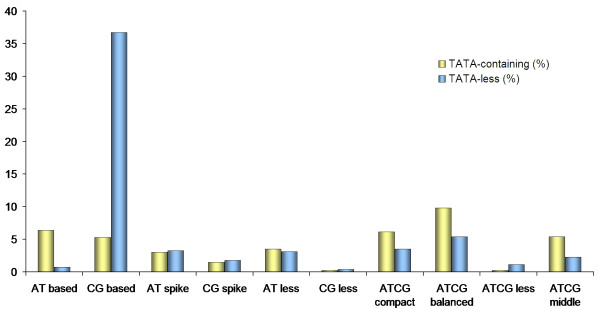
**TATA-less and TATA-containing correlations.** In each class, blue bars show the proportion of TATA-less promoters and light yellow bars show the proportion of TATA-containing promoters. Observations were made on a sample of 795 promoters, randomly selected from a collection of 8512 *Homo sapiens* promoters.

Several reports regarding *Homo sapiens* TATA-containing promoters seem to vary in different studies, depending on the number of promoters used [33]. An earlier study found 32% TATA-containing promoters from a set of ~1,000 genes [34]. More recent genome-wide studies show that only ~10% of human genes contain TATA-dependent promoters [20,35]. However, the EPD dataset (Additional file 1) has been cleared of redundant promoters that shared the same TSS. Accordingly, their promoter set has a much higher presence of known promoter elements, such as TATA or GC boxes. Using the EPD collection of 8,512 *Homo sapiens* promoters, we searched for TATA motifs in a sample of 795 promoter sequences. Of this collection, we found that ~41% were TATA-containing promoters (Additional file 2). TATA-containing promoter levels were higher in AT-based, AT-less, ATCG-compact, ATCG-balanced and ATCG-middle classes, whereas TATA-less promoter levels were higher in CG-based, AT-spike, CG-less and ATCG-less classes (Figure 5). More extreme differences between TATA-containing and TATA-less promoters were observed in CG-based (TATA-containing (5.28%), TATA-less (36.72%) and AT-based (TATA-containing (6.41%), TATA-less (0.75%) classes (Additional file 2).

### Transitional states

**Figure 6:**
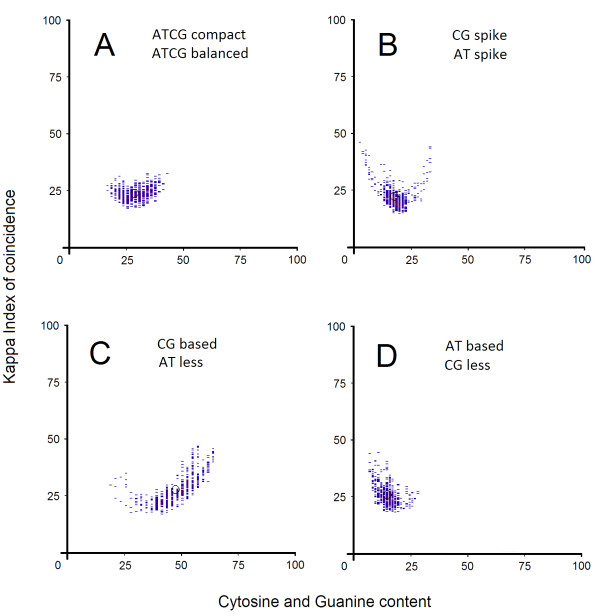
**Promoter patterns found in transitional states.** (**A**) MDH1B gene promoter found in a transitional state between ATCG-compact and ATCG-balanced class, (**B**) UFC1 gene promoter found in a transitional state between AT-spike and CG-spike class, (**C**) LRRN1 gene promoter found in a transitional state between AT-less and CG-based class and (**D**) PCDHB10 gene promoter found in a transitional state between AT-based and CG-less class.

Previous studies suggested that TATA-less and TATA-containing promoters have different chromatin structure [36-41]. Evolutionary, chromatin structure may influence the distribution of point mutations or other mutational events in the promoter sequence. A chromatin-dependent distribution of point mutations can lead to a gradual shift from a promoter class to another promoter class (ie. by disruption of poly(dA:dT) or poly(dC:dG) tracts in shorter elements), thus changing the predisposition for low or high levels of gene expression. Promoter patterns “trapped” in transitional states between classes may also perhaps indicate a change of their gene relationship towards other biological pathways. We have found intermediate states between these patterns which may suggest an evolutionary transition mechanism (Figure 6). Initially, the transition states were observed by our neural network (Additional file 3). All promoter patterns have been classified by the highest percentage of recognition for each class. Certain promoter patterns present similar percentages for two separate classes of promoters, indicating a potential inclusion in two classes simultaneously. Exact intermediate patterns are rare (sometimes even unique) and differ drastically from the majority of patterns (Figure 6). For instance, ATCG-balanced class appears to have several patterns with a transitional tendency to ATCG-compact class or vice versa (Figure 6A). These transitions are based on successive elimination/insertion of short poly(dA:dT) and poly(dC:dG) tracts. Another example is represented by a systematic reduction of short poly(dA:dT) tracts, which lead to a transition of AT-less promoters to CG-based class (Figure 6C). In contrast, a systematic reduction of short poly(dC:dG) tracts leads to a class transition from CG-less promoters to AT-based promoters (Figure 6D). From what we have witnessed, neither of these classes represent “end of the line” for these transitions since we observed intermediate patterns between all classes. Furthermore, we have observed varying degrees of difficulty of transition from one class to another. This difficulty is reflected in the number of promoters belonging to each class (Additional file 2). For example, CG-based and AT-based, AT-spike and CG-spike or AT-less and CG-less classes tend to form mirror pairs. These pairs of classes have the lowest probability to transit directly from one to another. The evidence for this claim is supported by a small number of intermediate patterns that we have found between these alleged pairs of classes. For instance, intermediate patterns between AT-spike and CG-spike promoters can have both long poly(dA:dT) and long poly(dC:dG) tracts, a sequence arrangement that is rarely encountered (Figure 6B). Consequently, we suggest that these direct transitions of promoters between pairs of classes may be caused by strong selection pressures conditioned by radical changes in the environment.

### Tissue-specificity in humans

**Figure 7:**
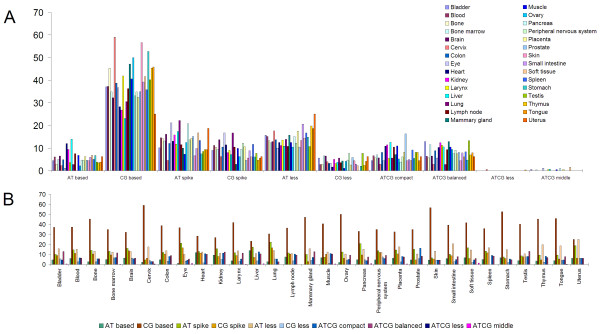
**Tissue-distribution frequencies for 2,369 human promoters.** Two visualization methods are used: (**A**) shows the distribution of 30 tissues for each class of promoters and section (**B**) shows the distribution of promoter classes in each tissue.

**Figure 8:**
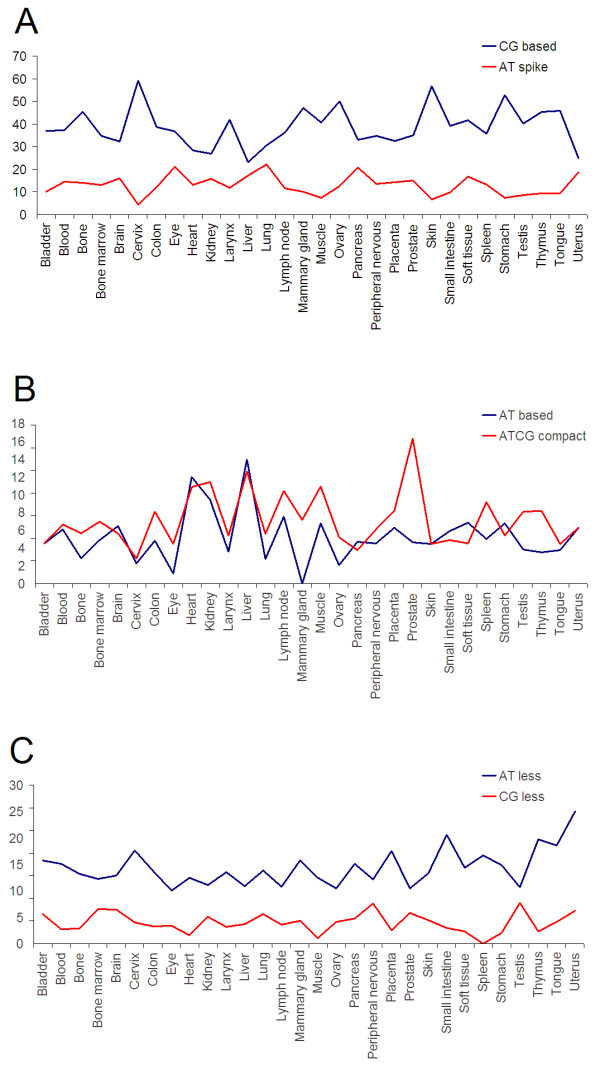
**An overall comparison between different promoter classes in each tissue.** (**A**) tendency for a complementarity relationship between CG based and AT spike classes, (**B**) tendency for direct proportionality relationship of AT based – ATCG compact classes. (**C**) a combined relationship between AT less – CG less classes, both of complementarity and direct proportionality.

Our general classification criterion allowed us to demonstrate compelling biological correlates between 2,369 tissue-specific genes (Figure 7A,B). Some of our observations are also based on previous studies that suggest direct correlations between short or long homopolymer tracts and certain levels of gene expression [42-46]. Indeed, we have also observed a constant presence of different homopolymer elements in these patterns, suggesting that different promoter classes (ie. CG-spike or AT-spike) indicate a predisposition for various levels of gene expression as well as for a distinct number of factors which trigger gene expression. Specific interaction clusters have been reported in the past, such as muscle and heart or kidney and liver clusters [30]. We show some additional interaction groups, both between promoter classes and within each promoter class. In addition to these groups, the tissue order from each class further reflects the significance of the observed interactions (Additional file 4). The highlights of our observations include:

1. CG-based promoters have the highest percentage of occurrence (37.59%) and appear to be TATA-less class correspondents which tend to be associated with “housekeeping” genes. CG-based promoters are not only the most common but as expected they show the highest levels in all tissues. The first six tissues in which CG-based promoters have the highest percentages are cervix, skin, stomach, ovary, mammary gland and tongue (Additional file 4: Figure S10B online).

2. AT-based promoters (5.25%) are present in all tissues but are absent from the mammary gland. The first six tissues in which AT-based promoters have the highest percentages are liver, heart, kidney, lymph node, soft tissue and muscle. This order coincides with the first six tissues in which ATCG-compact promoters have the highest percentages, namely in prostate, liver, kidney, muscle, heart and lymph node. Equally curious, the last six tissues in which CG-based promoters have the lowest percentages are liver, uterus, kidney, heart, lung and brain (Additional file 4: Figure S10G and Figure S 7B online). This implies a special relationship between CG-based and AT-based promoters because their proportions seem to indicate an almost antagonistic activity which may suggests an involvement of these promoters in some metabolic processes. Nevertheless, the relationship between CG-based promoters and other classes of promoters in these tissues seems to conceal more than a simplistic association with the housekeeping genes.

3. AT-less promoters (14.36%) are overestimated in uterus while CG-less and ATCG-balanced promoters are overestimated in testis (Additional file 4: Figure S10E,F,H online).

4. CG-less promoters have an occurrence of 3.98% and are present in all tissues but they are absent from Spleen (Additional file 4: Figure S10F online).

5. There was no clear correlation regarding tissue order between AT-less and CG-less promoters. Nevertheless, we noticed that some tissues have a tendency to stay grouped, such as muscle and heart, stomach and soft tissue, larynx and colon, lymph node and liver or bone marrow and peripheral nervous system (Additional file 4: Figure S10E,F online). These groups may suggest a role of these promoters in simple feedback mechanisms among tissues responsible for maintaining homeostasis. Furthermore, the occurrence of short poly(dA:dT) tracts on short distances near TSS could also indicate an involvement of AT-less (and, by association, a complementary role for their CG-less counterpart) promoters in short term non-critical gene expression, which may strengthen our hypothesis regarding their physiological role. Moreover, in different tissues AT-less and CG-less percentages show a combined relationship of complementarity and proportionality (Figure 8C).

6. AT-spike promoters are found especially in tissues that require high levels of gene expression such as lung, eye, pancreas, uterus, liver, soft tissue, brain, kidney, prostate and blood. This tissue order and the presence of long poly(dA) or long poly(dT) tracts suggests an involvement of these promoters in survival mechanisms, possibly responsible for interactions with the environment.

7. CG-spike promoters also appear to be involved in survival mechanisms. These promoters are found in large numbers especially in tissues that need a short-term critical gene expression. This is supported by the order of the first seven tissues in which these promoters are most common, such as lung, eye, brain, peripheral nervous system, spleen, heart and blood, which also tend to have a high interaction with the environment (Additional file 4).

8. The proportions of CG-spike and AT-spike promoters seem to be similar in the first two tissues, namely in lung and eye. The occurrence of long poly(dA:dT) or tandem short poly(dA:dT) tracts on short distances (>30b) near TSS, could also indicate an involvement of AT-spike and CG-spike promoters in short term critical gene expression.

 The frequency of AT-spike promoters (13.02%) exceeds that of GC-spike promoters (8.93%) but indicate proportional relative values in most tissues. Exceptions are tissues from cervix and muscle where the number of CG-spike promoters surpasses the number of AT-spike promoters (Additional file 4).

10. The percentage of occurrences between CG-based and AT-spike promoters appears to be relative and nearly complementary in all tissues (Figure 8A). Interestingly, the last two tissues in which AT-spike promoters have the lowest percentages and the first two tissues in which CG-based promoters have the highest percentages are cervix and skin (Additional file 4: Figure S10C,B).

11. The proportion of ATCG-compact and AT-less promoters seems to have similar values in tissues from kidney and lymph node whereas ATCG-compact and AT-based promoters appear to have similar values in bladder, skin and uterus (Figure 8B). ATCG-compact promoters tend to exhibit equal values in some tissues such as liver and kidney, brain and bone, heart and muscle. Interestingly, AT-based promoters show also equal values in these tissues but different than those found for ATCG-compact promoters (Additional file 4).

12. There was no clear correlation regarding the tissue order between ATCG-balanced and ATCG-compact promoters. However, ATCG-balanced and ATCG-compact promoters seem to have almost equal percentages in about 16 tissues. Both of these classes have the closest values in blood, bone, brain, cervix, colon, heart, muscle, skin and uterus (Additional file 4).

13. ATCG-less promoters are rare (0.03%) and are even more enigmatic since they are mainly represented in cervix and tongue (Additional file 4: Figure S10I online). In humans, from a total of 8,512 promoter sequences the percentage of ATCG-less promoters it is close to 1.08% whereas their appearances among 2,369 promoters of tissue-specific genes it is almost 0.03%. These results are not consistent with ATCG-less expected frequency of 0.3%, which may suggest that most of their genes are silent (Additional file 4).

14. ATCG-middle promoters are present only in nine of the thirty tissues, namely in soft tissue, eye, pancreas, liver, placenta, bladder, muscle, larynx and bone marrow (Additional file 4: Figure S10J online). However, in humans, from a total of 8,512 promoter sequences the percentage of ATCG-middle promoters it is close to 1.05%. Nevertheless, from 2,369 promoters of tissue-specific genes the observed frequency is close to 0.22% whereas their expected frequency is 0.29%, which suggests that some of their genes are also silent. The difference between expected and observed frequencies and an overall low occurrence of genes containing ATCG-middle and ATCG-less promoters may suggest their involvement in anatomical development and in some other cell-related cycles. This observation is supported by several tests performed on promoters from HOX gene family, namely HOXA and HOXB. These genes are represented mostly by patterns showing ATCG-middle characteristics. (Additional file 5: Figure S13A-E and Figure S14A-E online). A more broad analysis involving expected and observed frequencies for all classes of promoters is presented in our Additional file 6.

## Discussion

**Figure 9:**
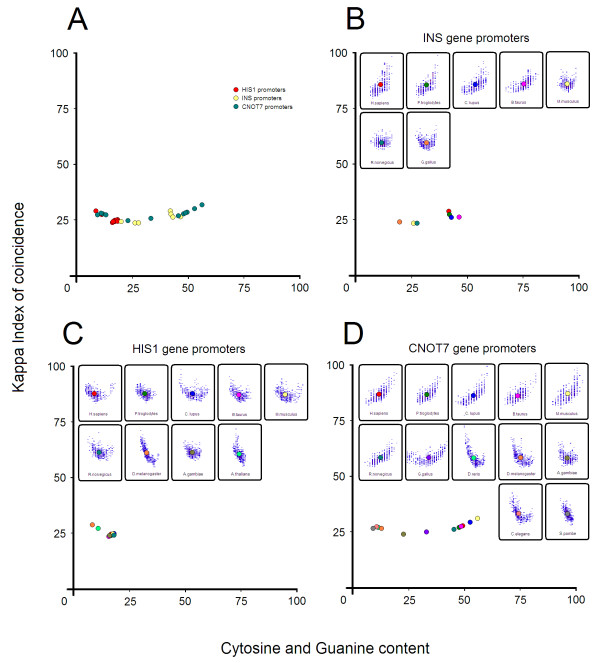
**Distribution across promoters of orthologous genes.** (**A**) overlapping distribution of orthologous promoters from INS (yellow circles), HIS1 (red circles) and CNOT7 gene (green circles), (**B**) distribution of orthologous promoters from INS genes, (**C**) distribution of orthologous promoters from HIS1 genes, (**D**) distribution of orthologous promoters from CNOT7 genes. Each circle represents the center of weight from a promoter pattern and the circle color is associated with a corresponding species.

Generally, both EPD and PlantProm DB define the TSS as the furthest 5 position in the genome which can be aligned with the 5 end of a cDNA from the corresponding gene [25]. However, many human genes are transcribed from multiple promoters, often involving alternative first exons. EPD considers the most frequent cDNA 5 end as the TSS and applies a specialized algorithm to discover multiple promoters for a given gene, whereas PlantProm DB contains plant promoters based on published TSS mapping data [27]. Using a smaller number of promoters from EPD, we have also made an analysis for *Bos taurus*, *Gallus gallus*, *Mus musculus*, *Rattus norvegicus* and *Xenopus laevis* which showed a distribution close to that of *Homo sapiens* (Additional file 7: Figure S15A-E online). Therefore, promoter distributions (Figure 3A) seem to be characteristic for all vertebrates rather than a special property of human promoters. However, more significant differences were especially observed in *Gallus gallus*, where the average Kappa IC values exceed that of other vertebrates (Additional file 7: Figure S15B online). On a visual inspection, promoter patterns from *Arabidopsis thaliana* and *Drosophila melanogaster* have a more narrow shape than those from *Oryza sativa* and *Homo sapiens*, which suggests a different distribution of point mutations between these species, resulting perhaps from a difference in nucleosome organization. Furthermore, in our experiments we have found that an even distribution of mutations across different promoter sequences fails to change the shape of their patterns, which strengthened our hypothesis (Additional file 8: Figure S16A-D). We also noticed that even for shorter promoter sequences (ie. Arabidopsis - PlantProm DB - 251b promoter sequences), promoter patterns retain their properties. Curiously, sliding windows situated at greater distances from TSS do not seem to make a crucial difference in the pattern shape. The majority of defining characteristics seem to be close to TSS. We further made a distribution across promoters of known orthologous genes (Figure 9A-D). We used HomoloGene [47] to extract 500 bp genomic regions upstream of INS orthologous genes from 7 species, HIS1 orthologous genes from 9 species and CNOT7 orthologous genes from 12 species (Additional file 1). We confronted these genomic regions with EPD promoters in order to ensure their accuracy. As expected, their distribution (Figure 9A) retained the same species-specific boundaries (Figure 3A-D) and their promoter patterns comply with existing phylogenetic relationships (Figure 9B-D). For tests performed on human tissues we used a list of genes from TiGER (Tissue-specific Gene Expression). For each gene in this list we searched the corresponding promoter in the Eukaryotic Promoter Database. It was shown that these classes of promoters are preferentially present in certain tissues while other classes of promoters are present in all tissues (Additional file 4: Figure S11 online). Only six out of ten classes of promoters are present in all 30 tissues (Figure 7). Moreover, it was noted that in certain tissues some classes of promoters can occur in a complementary manner, whereas other classes of promoters can appear in competition (Additional file 4: Figure S12A-AS online). On comparisons made between three promoter classes, other types of promoter relations can unfold. For instance, in tissues from brain, eye or lung, the values for AT-less and AT-spike promoters appear to exhibit a relative complementarity to those from muscle, whereas the number of CG-spike promoters remains proportional to their relative values (Figure 7B). These parallel behaviors and the tissue-preferential distribution of these promoters suggest that certain promoter classes are preferred for specific biological functions. Therefore, these promoter patterns seem to explain the relationship between their genes in certain biological pathways rather than their gene-specific function. This observation implies that promoters located in transitional states may perhaps reflect signatures of some of the latest evolutionary changes of a species. Biological tissues are complex structures, containing different cell types. Accordingly, ‘tissue specific’ stands as a relative term and does not imply that a particular gene is expressed only in a specific tissue or cell type. To determine whether a gene is predominantly expressed in a certain tissue, TiGER defined the Expression Enrichment (EE) as the ratio between observed expression level in that tissue versus averaged expression level across 30 tissues. They further defined a gene as ‘tissue specific’ if it had an EE in a particular tissue larger than 5 and a P-value <10^-3.5^[30]. Although “tissue specific” is a relative term and refers to genes predominantly expressed in different tissues, the fundamental tissue-tissue interactions are reflected in our promoter pattern analysis (Additional file 4).

## Conclusions

A comparative analysis was undertaken for 20,586 promoters from the *Arabidopsis thaliana*, *Drosophila melanogaster*, *Homo sapiens* and *Oryza sativa* (Additional file 2), and an analysis based on tissue-specific gene expression profiles in humans (Additional file 4). Following the analysis, 10 general classes of promoters have emerged. We used promoter sequences from two databases - the Eukaryotic Promoter Database and PlantProm Database. We showed that existing methods used in cryptography, such as Kappa Index of Coincidence, can be adapted for many types of analysis in molecular genetics, perhaps to highlight certain new features of DNA sequences. Our supplemental data files allow re-analysis of our data. We also provide an animation that displays several hundred promoter patterns in succession and ordered according to their class (Additional file 9). We consider a possible subdivision of these promoter patterns in subclasses, between 2 up to 4 subclasses for each major class. Furthermore, our observations suggest the existence of a network between these promoter classes. In the near future we wish to merge the information related to these classes of promoters with other available data in gene regulatory networks, in order to form a better understanding of the relationship between some genetic factors and their pathological implications.

## Methods

### Promoter datasets

The Eukaryotic Promoter Database and PlantProm Database provide a collection of eukaryotic promoters for which the transcription start site (TSS) has been determined experimentally (Additional file 1). We downloaded and tested 20,586 gene promoters from The Eukaryotic Promoter Database (6,649 gene promoters - *Oryza sativa*, 1,922 gene promoters - *Drosophila Melanogaster* and 8,512 gene promoters - *Homo sapiens*) and PlantProm Database (3,503 gene promoters - *Arabidopsis thaliana*). We were mainly interested in the regions flanking the putative TSS. From Eukaryotic Promoter Database we extracted promoter segments ranging from -499b to 100b, relative to the TSS. From PlantProm DB we used promoter segments ranging from 200 bp upstream and 51 bp downstream of the TSS.

### Tissue-specific datasets

We used a publicly available list of 6,534 tissue-specific gene names (under Tissue-Specific Genes based on Expressed Sequence Tags (ESTs)) from the TiGER database (gene names were sorted and redundancy was removed - Additional file 10) and we searched for their promoters in the Eukaryotic Promoter Database in which we found 2,369 promoters. We generated 2,369 promoter patterns and we sorted them in order to highlight their proportion in each tissue (Additional file 11).

### Promoter patterns

We used Visual Basic to develop a software program for promoter analysis - called PromKappa (Promoter analysis by Kappa), and a software program for sorting promoter patterns - called PromNN (Promoter analysis by Neural Network). The source code implementation of these programs are attached to our Additional file 3. Promoter patterns were generated by PromKappa program. We used sliding window approach to extract two types of values: Kappa IC and (C + G)%. A sliding window with a step of 1 and a window size of 30 nt, allowed us to detail the structure of known promoters. Kappa Index of Coincidence values were plotted on a graph against (C + G)% values, which form a recognizable pattern composed from clusters of various sizes on the Y-axis (Figure 1A-J). The X-coordinate of each point was represented by a (C + G)% value and the Y-coordinate was represented by a corresponding Kappa IC value. As can be expected, by using a large window size we obtained smooth promoter patterns, whereas a small window size generated sharp and distinguishable characteristics of promoters which have been easily categorized.

### Promoter analysis

We conducted three types of analysis. Initially, for each promoter sequence we generated a graph, representing a promoter pattern. In total, we generated 20,586 graphs (Additional file 12). These graphs were saved in BMP (Bitmap Image File) format and were sorted by their shape and density using a neural network. In the second analysis, the center of each pattern was plotted on a graph designed to show the distribution of promoters for each species. We used a color scheme to highlight the denser surfaces. Red areas represent clusters of similar promoters while blue areas represent unique or rare promoters (Figure 3A-D). For the third analysis, we measured the specificity of each promoter class among thirty tissues by using 2,369 promoters (Figure 7A,B).

### Pattern recognition and sorting

We have been able to demarcate promoter sequences into ten classes by using the maximum number (≥100) of appearances of similar promoter patterns. To determine the biological characteristics of promoter sequences, we have resorted to machine learning methods. All patterns were analyzed and sorted by PromNN, a pattern recognizer program using 93,264 artificial neurons and a single layer perceptron. It has the ability to learn patterns and classify them into specified classes. We used supervised learning to train the neural network by using 200 input patterns (20 of each class of promoters, 5 from each species - Additional file 13). PromNN recognized ten promoter classes and provided information about the match score and match percentage for each promoter pattern.

### Cytosine and guanine content

We extracted C + G values from each sliding window considering the nucleotide frequencies from the entire promoter sequence. In the first stage, to determine the (C + G)% content for the entire promoter sequence we used the formula:

$$CG_{\mathrm{TOT}}=\left(\frac{100}{{\left(A+T+C+G\right)}_{\mathrm{TOT}}}\right)\times {\left(C+G\right)}_{\mathrm{TOT}}$$

Where “TOT” (total) designates the promoter sequence. *CG*_*TOT*_ represents the percentage of cytosine and guanine of the entire promoter, *(A + T +* *C + G)*_*TOT*_ represents the sum of occurrences of A, T, C and G, and *(C + G)*_*TOT*_ represents the sum of occurrences of C and G. In the next stage we used the value of *CG*_*TOT*_ to calculate the (C + G)% content from the sliding window (SW):

$$CG_{\mathrm{SW}}=\left(\frac{CG_{\mathrm{TOT}}}{{\left(A+T+C+G\right)}_{\mathrm{SW}}}\right)\times {\left(C+G\right)}_{\mathrm{SW}}$$

Where *CG*_*SW*_ represents the percentage of cytosine and guanine from the sliding window. In this stage, *CG*_*SW*_ value is relative to *CG*_*TOT*_. The expression *(A + T +* *C + G)*_*TOT*_ represents the sum of occurrences of A, T, C and G from the sliding window sequence. *(C + G)*_*SW*_ represents the sum of C and G occurrences in the sliding window sequence. Nevertheless, in our implementation we also included the option to extract *CG*_*SW*_ values without considering *CG*_*TOT*_.

### Kappa Index of Coincidence

The Index of coincidence principle is based on letter frequency distributions and has been used for the analysis of natural-language plaintext in cryptanalysis. Kappa Index of Coincidence is a form of Index of Coincidence used for matching two text strings. Nevertheless, we managed to adapt Kappa IC for the analysis of a single DNA sequence. Here, Kappa IC is used for calculating the level of “randomization” of a DNA sequence. By extracting Kappa IC and C + G content from a sliding window we have been able to measure the localized values along each promoter sequence. Kappa IC is sensitive to various degrees of sequence organization such as simple sequence repeats (SSRs) or short tandem repeats (STRs). The formula for Kappa IC is shown below, where sequences *A* and *B* have the same length *N*. Only if an *A[i]* nucleotide from sequence A matches the *B[i]* correspondent from sequence *B*, then ∑ is incremented by 1.

$$Kappa_{\mathrm{IC}}=\frac{\sum_{i=1}^{N}\left[A_{i}=B_{i}\right]}{N/C}$$

With small changes, the same method for measuring the Index of Coincidence has been applied for only one sequence, in which the sequence was actually compared with itself, as shown below in the algorithm implementation.

function KIC(A)

T = 0

N = length(A) - 1

  for u = 1 to N

  B = A[u + 1] … A[N]

    for i = 1 to length(B)

    If A[i] = B[i] then C = C + 1

    next i

  T = T + (C / length(B) × 100)

  C = 0

next u

  IC = Round((T / N), 2)

end function

Where *N* is the length of the sliding window, *A* represents the sliding window content, *B* contains all variants of sequences generated from *A* (from *u + 1* to *N*), *C* counts the number of coincidences occurring between sequence *B* and sequence *A*, and *T* variable counts the total number of coincidences found between sequences of *B* and the sequence *A*.

## Competing interests

The authors declare that they have no competing interests.

## Authors’ contributions

PG conceived of the study and participated in its design and coordination. PG created the algorithms and the software used in the analysis. CIT carried out the assembly of promoter files and manually tested the correctness of each promoter sequence. PG and CIT participated in the promoter sequence analysis and drafted the manuscript. Both authors have verified the accuracy of the data and repeated the experiment independently. All authors read and approved the final manuscript.

## Supplementary Material

Additional file 1 **Promotor sequences.** A complete set of 20,586 gene promoters from The Eukaryotic Promoter Database (6,649 gene promoters - *Oryza sativa*, 1,922 gene promoters - *Drosophila Melanogaster* and 8,512 gene promoters - *Homo sapiens*) and PlantProm Database (3,503 gene promoters - *Arabidopsis thaliana*). Click here for file

Additional file 2 **Organism-specific data.** Comparative analysis undertaken for 20,586 promoters from *Arabidopsis thaliana*, *Drosophila melanogaster*, *Homo sapiens* and *Oryza sativa*. Click here for file

Additional file 3 **PromKappa and PromNN.** PromKappa (Promoter analysis by Kappa) software program used for promoter pattern generation and promoter analysis and PromNN (Promoter analysis by Neural Network) software program used for sorting promoter patterns. Click here for file

Additional file 4 **Tissue-specific data.** Promoter analysis in *Homo sapiens*, based on tissuespecific gene expression profiles. Click here for file

Additional file 5 **Observations for HOX genes.** Comparative analysis of HOX gene promoter patterns. Click here for file

Additional file 6 **Observed and expected frequencies.** Analysis involving expected and observed promoter frequencies based on Organism-specific and Tissue-specific data. Click here for file

Additional file 7 **Distribution in other species.** A secondary distribution of gene promoters in *Bos taurus*, *Gallus gallus*, *Mus musculus*, *Rattus norvegicus*, *Xenopus laevis* and *Zea mays*. Click here for file

Additional file 8 **DET1 promoter pattern.** Shows a simulation which highlights the promoter sequence resistance to random mutations. Click here for file

Additional file 9 **Promoter pattern animation.** Animation showing several hundred promoter patterns in succession and ordered according to their class. Click here for file

Additional file 10 **List of tissue-specific genes.** List of 6,534 tissue-specific gene names from TiGER database (gene names were sorted and redundancy was removed). Click here for file

Additional file 11 **Tissue-specific promoter patterns.** The complete set of 2,369 image-based promoter patterns used for tissue-specific analysis. Click here for file

Additional file 12 **Organism-specific promoter patterns.** The complete set of 20,597 image-based promoter patterns used for a comparative analysis of the four species taken into consideration. Click here for file

Additional file 13 **Gene promoters used for neural network training.** List of 918 image-based promoter patterns used for PromNN training. Click here for file
