# Supplementary material for: Eukaryotic genomes may exhibit up to 10 generic classes of gene promoters
Source: BMC Genomics. 2012 Sep 28;13:512. doi: 10.1186/1471-2164-13-512 (PMC3549790; doi:10.1186/1471-2164-13-512)
Supplement: Additional file 5 — Observations for HOX genes. Comparative analysis of HOX gene promoter patterns. [file 1471-2164-13-512-S5.doc]

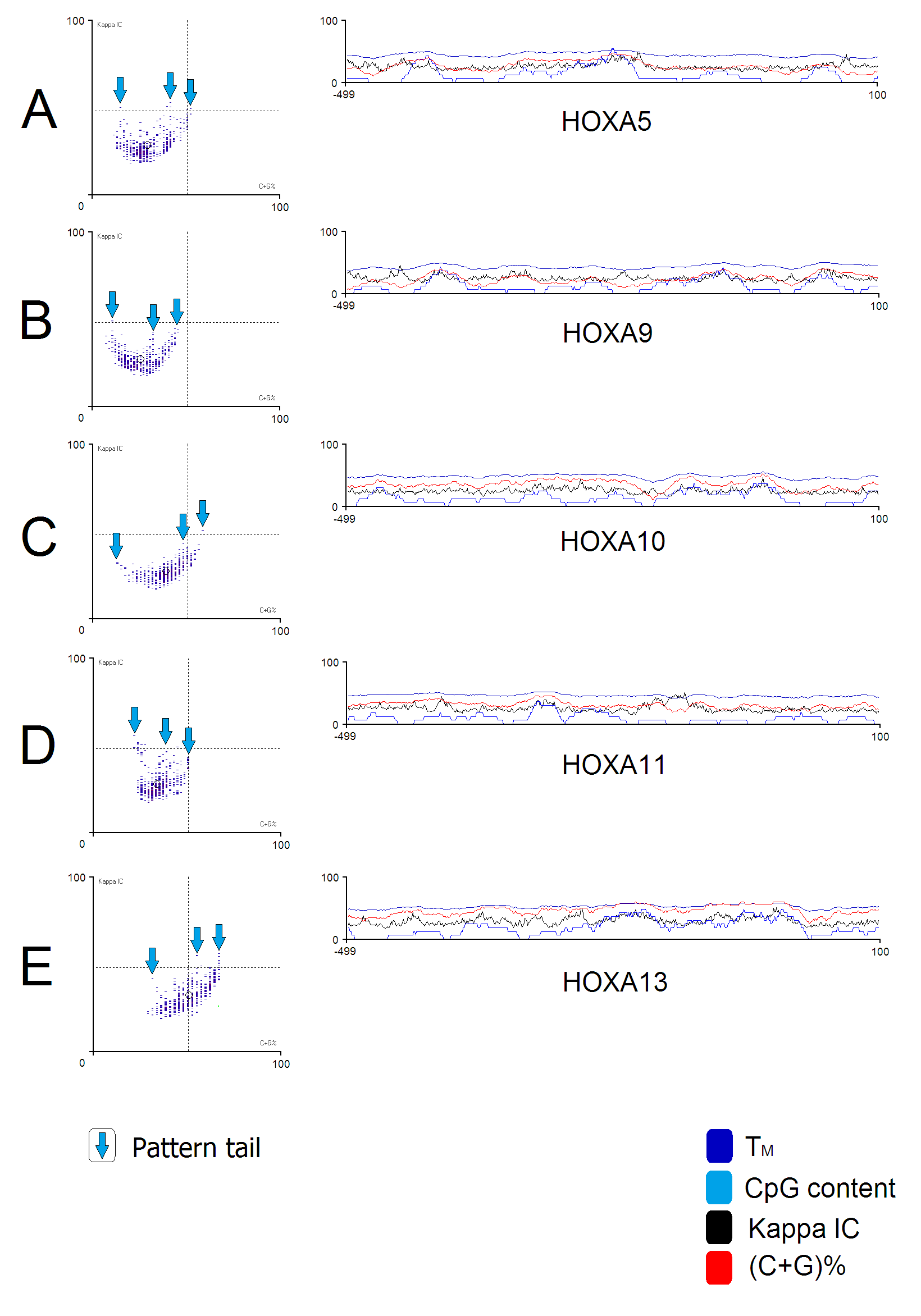


**Figure 13.** HOXA gene promoters. Most of HOX promoter patterns exhibit three vertical formations (tails) which are characteristic of ATCG-middle class. (A) HOXA5 gene contains an ATCG-middle promoter, (B) HOXA9 gene contains a classic ATCG-middle promoter, (C) HOXA10 gene also contains a classic ATCG-middle promoter, however, the middle tail contains sequences with a higher G+C content, (D) HOXA11 promoter pattern also contains a three tail shape, nevertheless, there is a noticeable tendency to AT-spike class, (E) HOXA13 promoter pattern shows ATCG-middle structural features but also a tendency towards CG-based class.


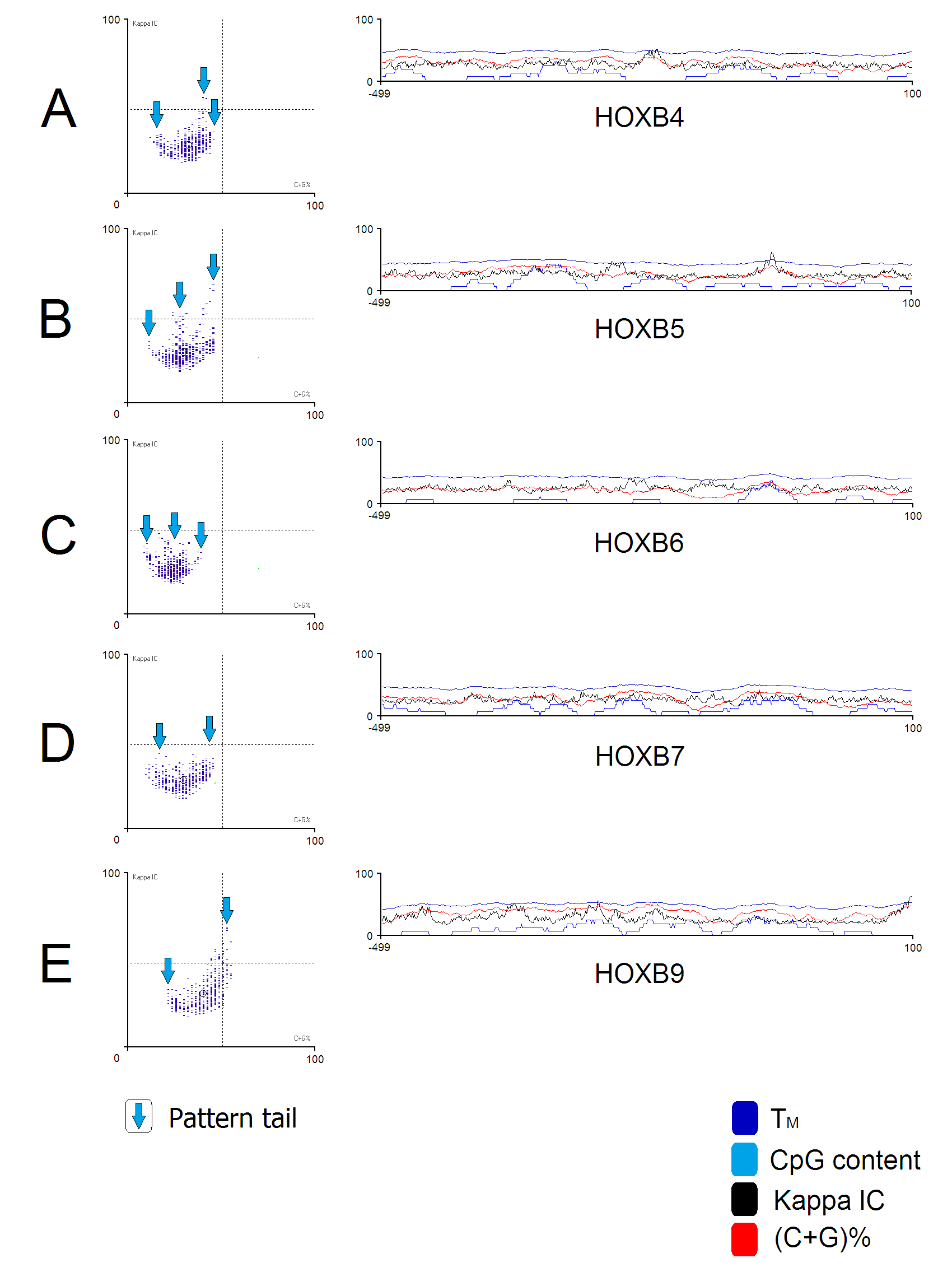


**Figure 14.** HOXB gene promoters. (A) HOXB4 promoter pattern showing ATCG-middle structural features with a tendency to CG-spike class, (B) HOXB5 gene appears to contain an ATCG-middle promoter with a tendency towards CG-spike class, (C) HOXB6 gene contains a classic ATCG-middle promoter, (D) HOXB7 gene contains a classic ATCG-balanced promoter, (E) HOXB9 gene contains a CG-based promoter.
