# Supplementary material for: Eukaryotic genomes may exhibit up to 10 generic classes of gene promoters
Source: BMC Genomics. 2012 Sep 28;13:512. doi: 10.1186/1471-2164-13-512 (PMC3549790; doi:10.1186/1471-2164-13-512)
Supplement: Additional file 7 — Distribution in other species. A secondary distribution of gene promoters in Bos taurus, Gallus gallus, Mus musculus, Rattus norvegicus, Xenopus laevis and Zea mays. [file 1471-2164-13-512-S7.doc]

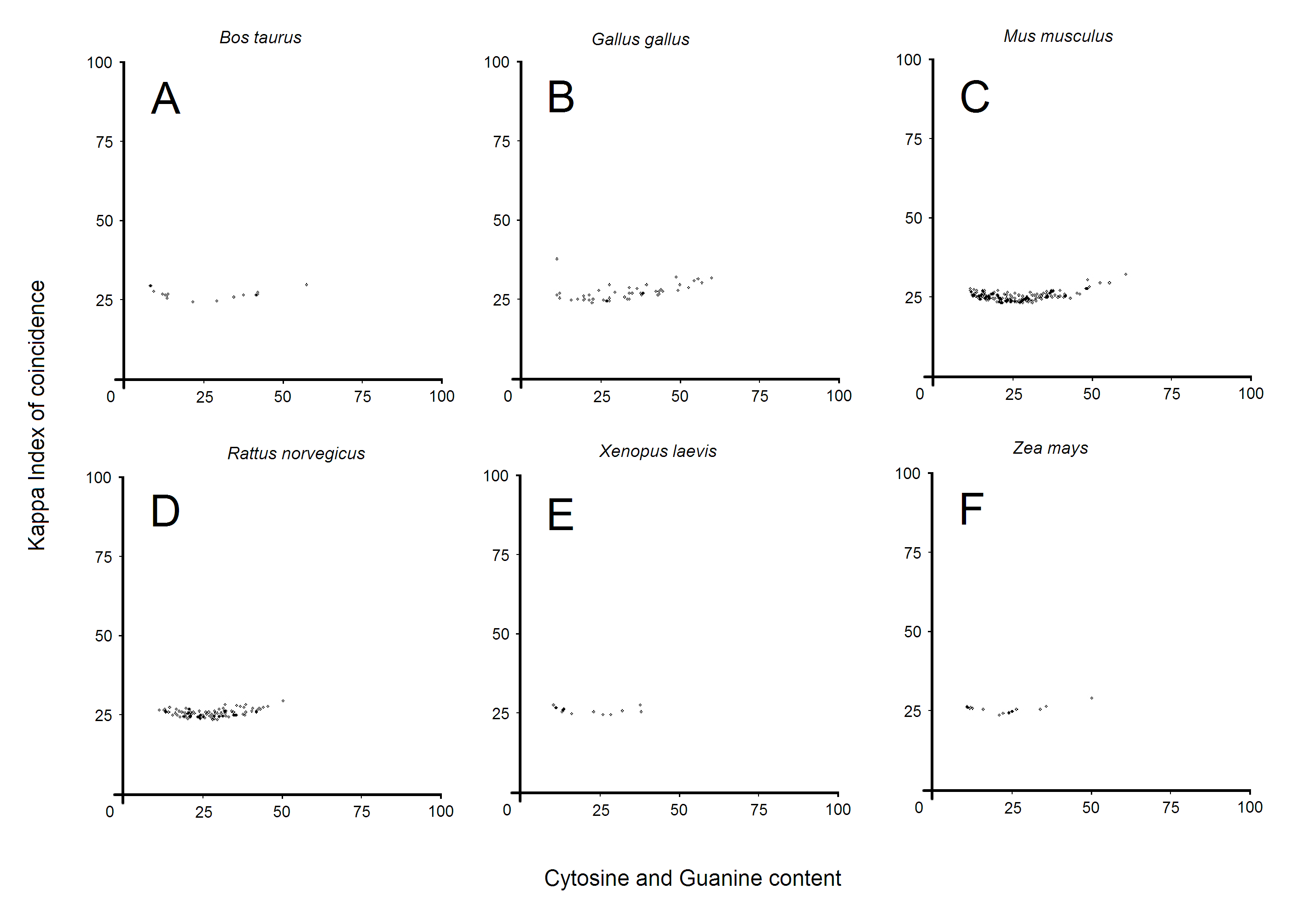


**Figure 15.** Promoter distributions for other six species. (A) *Bos taurus* (18 promoter sequences), (B) *Gallus gallus* (49 promoter sequences), (C) *Mus musculus* (147 promoter sequences), (D) *Rattus norvegicus* (107 promoter sequences), (E) *Xenopus laevis* (14 promoter sequences) and (F) *Zea mays* (18 promoter sequences). Each circle represents the center of weight from a promoter pattern.
