# Supplementary material for: Eukaryotic genomes may exhibit up to 10 generic classes of gene promoters
Source: BMC Genomics. 2012 Sep 28;13:512. doi: 10.1186/1471-2164-13-512 (PMC3549790; doi:10.1186/1471-2164-13-512)
Supplement: Additional file 8 — DET1 promoter pattern. Shows a simulation which highlights the promoter sequence resistance to random mutations. [file 1471-2164-13-512-S8.doc]

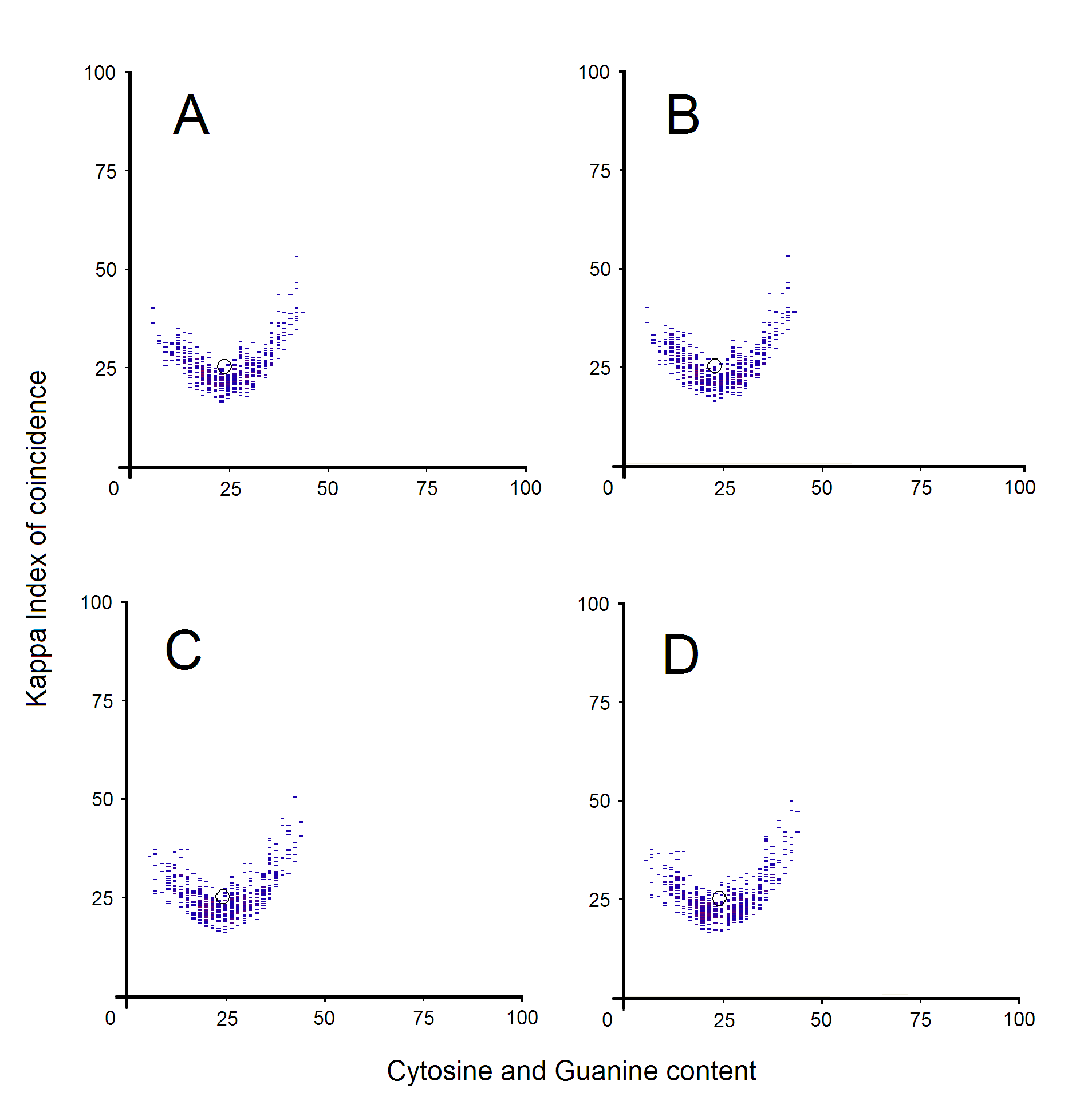


**Figure 16.** Promoter resistance to random mutations. (A) normal promoter patern of DET1 gene, (B) localizied homopolimer isertion, (C) isertion of random and equally intersprest nucleotides (10 periodicity), (D) isertion of random intersprest nucleotides.

DET1 promoter sequence (>EP 6758 (+) DET1_1; range -499 to 100) variants used to generate Figure 16 promoter patterns. Inserted nucleotides are shown in red.

1) Normal promoter patern of DET1 gene (Figure 16A):

AGCCATCCTAAGGGGTATGAAGTAGTATCTCATTGTTTTGATTTGCATTTCTCTGATAATTAATGATGTTGAAGGTCTTTTTGTTAGGGAAGCAGGAGCCTACGAGAGCCAGAGTGACATCATTTTAAAATCAACTCCATCTTAAAACTAGCAAGGCAAATTCCCTGTTAGTCAGAGATGATCAATACAATCTCAGTCTTCAAGGTGCTCGCCATCTAGAAGATAACAGAGTAGGAAGTTTCTCAGCATCTAATCAATAGGTGAAAAATTAATTCTATTCATATTTTTTCAAATGTGCCCAGCAGGTGCAGAGACACTGCCATTGGGACTCACAGAGATTGCCAGGTTAGGTGCCATTTTAATTTTTCCTTCGGAGCCATGGAGCCTCTACGGCTTCGATCCTTCCTCCGGGCACAAGGGTGCGCTGTACCTGTCTCTTAGTGTTGGCTGAACAGTGCCGTGCTCTCCCCTACTTCCCTTTTTGCTGGGCCGCAGTTCCGGTGGCTGGCGGAAACGGGAACGTGCAGCCGCGGGTGCAGGAGTCCTGGGGCATGGCGGGGGCGGGGCAGGGGGAGGCGCGCACAGAACAGGCTGGGGCAT

2) Localizied homopolimer isertion (Figure 16B):

AGCCATCCTAAGGGGTATGAAGTAGTATCTCATTGTTTTGATTTGCATTTCTCTGATAATTAATGATGTTGAAGGTCTTTTTGTTAGGGAAGCAGGAGCCTACGAGAGCCAGAGTGACATCATTTTAAAATCAACTCCATCTTAAAACTAGCAAGGCAAAAAAAAATTTTTTTGGGGGGAAAAAATTCCCTGTTAGTCAGAGATGATCAATACAATCTCAGTCTTCAAGGTGCTCGCCATCTAGAAGATAACAGAGTAGGAAGTTTCTCAGCATCTAATCAATAGGTGAAAAATTAATTCTATTCATATTTTTTCAAATGTGCCCAGCAGGTGCAGAGACACTGCCATTGGGACTCACAGAGATTGCCAGGTTAGGTGCCATTTTAATTTTTCCTTCGGAGCCATGGAGCCTCTACGGCTTCGATCCTTCCTCCGGGCACAAGGGTGCGCTGTACCTGTCTCTTAGTGTTGGCTGAACAGTGCCGTGCTCTCCCCTACTTCCCTTTTTGCTGGGCCGCAGTTCCGGTGGCTGGCGGAAACGGGAACGTGCAGCCGCGGGTGCAGGAGTCCTGGGGCATGGCGGGGGCGGGGCAGGGGGAGGCGCGCACAGAACAGGCTGGGGCAT

3) Isertion of random and equally intersprest nucleotides (Figure 16C):

AGCCATCCTACAGGGGTATGAAAGTAGTATCTGCATTGTTTTGTATTTGCATTTTCTCTGATAATATAATGATGTTGGAAGGTCTTTCTTGTTAGGGACAGCAGGAGCCATACGAGAGCCGAGAGTGACATGCATTTTAAAATTCAACTCCATTCTTAAAACTACGCAAGGCAAAATTCCCTGTTAAGTCAGAGATGGATCAATACAACTCTCAGTCTTCCAAGGTGCTCAGCCATCTAGATAGATAACAGATGTAGGAAGTTGTCTCAGCATCGTAATCAATAGCGTGAAAAATTAAATTCTATTCTATATTTTTTCCAAATGTGCCCAAGCAGGTGCAGGAGACACTGCGCATTGGGACTGCACAGAGATTGGCCAGGTTAGGGTGCCATTTTTAATTTTTCCTCTCGGAGCCATAGGAGCCTCTATCGGCTTCGATGCCTTCCTCCGCGGCACAAGGGGTGCGCTGTACTCTGTCTCTTAGGTGTTGGCTGTAACAGTGCCGATGCTCTCCCCCTACTTCCCTTATTTGCTGGGCCCGCAGTTCCGGGTGGCTGGCGTGAAACGGGAAGCGTGCAGCCGGCGGGTGCAGGGAGTCCTGGGGACATGGCGGGGGGCGGGGCAGGCGGGAGGCGCGACACAGAACAGGGCTGGGGCATT

4) Isertion of random intersprest nucleotides (Figure 16D):

AGCCATCCTACAGGGGTATGAAGTAGATATCTGCATTGTTTTGTATTTGCATTTTCTCTGATAATATAATGGATGTTGAAGGTCTTTTTGTCTAGGGACAGCAGGAGCCATACGAGAGCCGAGAGTGACATCATGTTTAAAATCAACTCCATTCTTTAAAACTAGCAACGGCAAAATTCCCTGTATAGTCAGAGATGATCAGATACAATCTCCAGTCTTCCAAGGTGCTCAGCCATCTAGATAGATAACAGATGTAGGAAGTTTCTCAGCATCGTAATGCAATACGGTGAAAAAATTAATTCTATTTCATATTTTTTCCAAATGTGACCCAGCAGGGTGCAGAGACACTGCCAGTTGGGACTCACAGAGATGTGGCCAGGTTAGGTGCGCATTTTATATTTTTCCTCTCGGAGCCAATGGAGCCTTCTACGGCTTCGAGTCCTTCCTCCGGGCCACAAGGGTGCGGCTGTACTCTGTCTGCTTAGTGTTTGGCTGAACAGTGCACGTGCTCTCCCCCTACTTCCCTTTTTGCTAGGGCCGCACGTTCCGGTGGCGTGGCGGAAATCGGGAACGTGGCAGCCGCGGGGTGCAGGGAGTCCTGGAGGCATGGCGGGGGGCGGGGCCAGGGGGAGGCGACGCACAGGAACAGGCTGGGGTCAT
